# Comparative Transcriptomics Unveils Pathogen-Specific mTOR Pathway Modulation in *Monochamus alternatus* Infected with Entomopathogenic Fungi

**DOI:** 10.3390/insects16101006

**Published:** 2025-09-28

**Authors:** Haoran Guan, Jinghong He, Chuanyu Zhang, Ruiyang Shan, Haoyuan Chen, Tong Wu, Qin Sun, Liqiong Zeng, Fangfang Zhan, Yu Fang, Gaoping Qu, Chentao Lin, Shouping Cai, Jun Su

**Affiliations:** 1 Basic Forestry and Proteomics Research Center, Fujian Provincial Key Laboratory of Haixia Applied Plant Systems Biology, School of Future Technology, Fujian Agriculture and Forestry University, Fuzhou 350002, China; haoranguanfafu@foxmail.com (H.G.); 18828359901@163.com (J.H.); zcy198610@163.com (C.Z.); wukices@163.com (H.C.); wt20000427@163.com (T.W.); qugp68@163.com (G.Q.); chentaolin163@163.com (C.L.); 2 Fujian Academy of Forestry Sciences, Fuzhou 350012, China; 13313777245@189.cn (L.Z.); zff007@126.com (F.Z.); 3 Tea Research Institute, Fujian Academy of Agricultural Sciences, Fujian Branch of National Center for Tea Improvement, Fuzhou 350013, China; fjnkycys@163.com; 4 No. 29 Middle School, Taiyuan 030024, China; mylovelyniuer@163.com; 5 Institute of Soil Fertilizer, Fujian Academy of Agricultural Sciences, Fuzhou 350003, China; fangyu1106@163.com

**Keywords:** pine wilt disease, *Monochamus alternatus*, *Beauveria bassiana*, *Metarhizium anisopliae*, dual RNA-seq, mTOR pathway

## Abstract

Pine wilt disease (PWD), transmitted by *Monochamus alternatus* (JPS), poses a severe threat to global pine forests. Although the entomopathogenic fungi *Beauveria bassiana* (Bb) and *Metarhizium anisopliae* (Ma) represent environmentally friendly biocontrol alternatives, their practical application is limited by inconsistent field performance and an incomplete understanding of host–pathogen interactions. We employed dual RNA-seq at the critical 48 h infection time point to systematically compare the transcriptional responses between JPS and Bb/Ma during infection. Key findings revealed distinct infection strategies: Bb preferentially induced autophagy pathways and modulated host carbohydrate metabolism to facilitate nutrient acquisition, triggering corresponding tissue degradation responses in JPS. In contrast, Ma primarily co-opted host amino acid and sugar metabolic pathways for biosynthetic processes, eliciting a stronger immune defense activation in JPS. Notably, the mTOR signaling pathway was identified as a key regulator of the differential host responses to various entomopathogenic fungi. Further functional validation-specifically, the application of a chemical inhibitor and RNAi targeting mTOR in JPS-confirmed that mTOR inhibition selectively enhanced Bb-induced mortality in JPS without affecting Ma virulence. Our findings reveal the molecular determinants of host–pathogen specificity in PWD biological control and indicate that mTOR regulation could serve as an effective strategy to improve fungal pesticide performance.

## Simple Summary

Pine wilt disease is devastating pine forests worldwide and is spread by beetles called *Monochamus alternatus*. Two environmentally friendly fungi, *Beauveria bassiana* and *Metarhizium anisopliae*, are pathogenic to these beetles, but their success in the field has been inconsistent. To elucidate the divergent mechanisms underlying fungal pathogenicity, we infected beetle larvae with each fungus and analyzed gene activity after 48 h. *Beauveria bassiana* triggered the mTOR-mediated autophagy-apoptosis regulatory network in beetles, while *Metarhizium anisopliae* relied on beetle nutrients for rapid growth. When we blocked the mTOR system with drugs or gene silencing, *Beauveria bassiana* was pathogenic to significantly more beetles, while *Metarhizium anisopliae* remained unaffected. This demonstrates that mTOR is crucial for beetles to defend against *Beauveria bassiana*. Our discovery offers a simple way to boost these fungi as safe pest-control tools: adding mTOR blockers only when using *Beauveria bassiana*-based products could make them more effective, reduce chemical pesticide use, and help protect pine forests and the environment.

## 1. Introduction

Pine wilt disease (PWD), caused by the pine wood nematode (*Bursaphelenchus xylophilus*; PWN) and transmitted primarily by the Japanese pine sawyer (*Monochamus alternatus*; JPS) in southern China, poses a formidable threat to global forest ecosystems due to its broad host range, rapid spread, and limited control options. [1,2]. Current strategies for managing JPS involve physical barriers [3], chemical pesticides [4], and biological controls approaches [5], among which entomopathogenic fungi (EPF) have gained increasing attention as particularly promising agents. Notably, *Beauveria bassiana* (Bb) and *Metarhizium anisopliae* (Ma) dominate 67.8% of commercial EPF formulations globally, offering advantages such as broad host specificity, environmental persistence, and high virulence [6,7]. Despite their potential as sustainable alternatives to chemical pesticides [8,9], their field efficacy remains inconsistent due to environmental variability and biological constraints [10].

The performance of these EPF species exhibits distinct environmental dependencies: Ma virulence correlates with elevation, pH, and micronutrient availability (P, Cu), while Bb efficacy is modulated by soil organic carbon, nitrogen, and potassium levels [11,12]. Crucially, their delayed action—attributable to required infection periods—limits rapid outbreak response, and strain-specific variability further complicates field applications [13]. To overcome these limitations, three strategic approaches have emerged: (i) formulation optimization [14], (ii) selection of high-virulence strains [15], and (iii) genetic engineering for enhanced pathogenicity and environmental resilience [16,17]. While genetic modification shows particular promise for accelerating virulence [18], critical knowledge gaps persist regarding: (1) molecular dialogs between Bb/Ma and JPS, (2) virulence factor-host gene interplay, and (3) dynamic mechanisms of fungal pathogenesis and host immune evasion [19,20].

At the mechanistic level, Bb and Ma employ multifaceted infection strategies. Bb utilizes a phosphorylation cascade (BbSte12-Bbmpk1) for cuticle penetration [21,22], supported by enzymatic arsenals including subtilisin-like proteases and chitinases [23,24]. Concurrently, it secretes laccase-2 to neutralize host oxidative defenses [25]. Ma employs distinct tactics, leveraging the LaeA regulator to activate destruxin production, which disrupts calcium signaling and suppresses Toll/Imd immune pathways [26,27]. Despite these advances, real-time analyses of JPS-fungal interactions remain scarce, particularly regarding transcriptional dynamics during infection [28,29].

To address these gaps, we employed dual RNA-seq [30] to map transcriptional networks during JPS infection by Bb and Ma, revealing a novel signaling pathway governing differential host responses. This work provides genetic targets for the precision engineering of EPF virulence and a molecular framework for optimizing biological control strategies against PWD.

## 2. Materials and Methods

### 2.1. Sample Collection

The *Beauveria bassiana* (BbFZ-51) and *Metarhizium anisopliae* (MaZP-01) strain used in this study was isolated and preserved by the Fujian Academy of Forestry Sciences, Fuzhou. The Bb and Ma mycelia were incubated for 14 d at 25 ± 1 °C and 50% humidity with a 12 L:12 D photoperiod in a Biochemical Incubator (SPX-300BSH-II, Shanghai Xinmiao Experimental Equipment Co., Ltd., Shanghai, China) on potato dextrose agar (PDA) media (Cat No. P8931, Solarbio, Beijing, China).

The JPS third-instar larvae were collected from individual pine trees from a 20-year-old Masson pine (*Pinus massoniana*) forests in Quanzhou (24°56′32″ N, 118°36′53″ E), Fujian, China. These collected larvae were then cultured in round plastic vials (7.0 cm in height × 5.0 cm in diameter) and placed in a completely dark condition in a incubator maintained at 30 °C and 50% humidity. They were reared on the pine sawdust obtained from the aforementioned Masson pine forest, until they reached the third-instar stage, at which point they could be used for further assays.

### 2.2. Fungus Inoculate onto JPS

Conidia of Bb or Ma were scraped from the colony surfaces and used to prepare a spore suspension with 0.03% Tween-80 (Cat No. T8360, Solarbio, Beijing, China) solution to a concentration of 10^9^ spores/mL. Third-instar larvae were washed up to 10 times in distilled water to remove debris. Then the larvae were immersed in the spore suspension for 15 s. Same amount of 0.03% Tween-80 solution was served as negative control. To eliminate experimental errors caused by the status differences between individual JPS larvae, a blank control group was established, which only involved washing without any further treatment. Each spore suspension and control treatment including 30 larvae. After inoculation, the larvae of BbJPS (JPS infected by Bb) and MaJPS (JPS infected by Ma) were placed in an incubator at 30 °C and 50% humidity in the dark.

### 2.3. Quantification of EPF Pathogenicity

After inoculation, all treated JPS larvae were examined at 12 h intervals until the conclusion of the experiment. The inspection focused on the presence of infection-related symptoms in the larvae, such as alterations in invasion spots and diminished vitality, as well as on mortality. Larvae were deemed dead if it exhibited no physiological activity and failed to respond to tactile stimulation. Dead larvae were individually transferred to a humid chamber for cultivation, facilitating fungal conidiogenesis. Fungal infection was confirmed as the cause of death if fungal growth and conidial production were observed following cultivation. Pathogenicity was assessed by calculating the mortality rate and determining the relative fungal expression levels. Mortality rate is calculated as follows: mortality rate (%) = treatment group mortality rate (%) − blank control group mortality rate (%). The relative fungal colonization levels were determined by quantitative analysis using quantitative polymerase chain reaction (qPCR).

Total RNA extraction was performed in triplicate for 24 and 48 h post-infection (hpi) JPS infected by Bb or Ma, a total of 12 samples were obtained using the Total RNA Extraction Kit (Cat No. R4151-02, Magen, Guangzhou, China) and sent for sequencing.

### 2.4. Dual RNA-Seq and Data Analysis

Total RNA was extracted using TRIzol reagent (Cat No. 15596026, Invitrogen, Carlsbad, CA, USA) and treated with DNase I (Cat No. RR047A, Takara, Kusatsu, Japan). RNA integrity and quantity were assessed using agarose gel electrophoresis, an Agilent 2100 Bioanalyzer (Agilent Technologies, Santa Clara, CA, USA), and a NanoDrop spectrophotometer (Thermo Fisher Scientific, Waltham, MA, USA). Sequencing libraries were prepared from 1.5 μg of total RNA per sample using the NEBNext^®^ Ultra™ RNA Library Prep Kit (Cat No. E7530, New England Biolabs, Ipswich, MA, USA) following the manufacturer’s protocol, including mRNA purification, fragmentation, cDNA synthesis, size selection (~200–250 bp), and PCR amplification. Library quality was verified on an Agilent 2100 Bioanalyzer. Paired-end sequencing (150 bp) was performed on an Illumina Novaseq 6000 platform (BGI) by the Beijing Allwegene Technology Company Limited (Beijing, China). The dataset generated can be found in the Sequence Read Archive (SRA) with the accession number PRJNA1286093.

Due to the Dual RNA-seq nature (host and pathogen), raw reads were first classified by mapping to the respective reference genomes: *Monochamus alternatus* (GCA_037114965, NCBI BioProject PRJNA1084890, 2024), *Beauveria bassiana* strain BbFZ-51 (GCA_044509585) [31], and *Metarhizium anisopliae* strain MaZP-01 (GCA_001765725) [32]. Raw fastq files were first processed with Trimmomatic v0.39 [33] to remove adapter sequences, reads containing poly-N, and low-quality bases (SLIDINGWINDOW:4:20 LEADING:3 TRAILING:3 MINLEN:50). Quality metrics (Q20, Q30, GC-content and duplication level) were subsequently calculated with fastp v0.20.0 [34]. All procedures followed the standard eukaryotic-reference-transcriptome pipeline of Allwegene GeneTech Co., Ltd. (Beijing, China). The quality control of raw reads for BbJPS, MaJPS, Bb, and Ma is shown in Figures S1–S4 and Table S1, respectively. Clean reads were aligned to the reference genome using HISAT2 v2.1.0 [35] with strand-specific default parameters. Alignment files were sorted and PCR duplicates were marked with Picard-tools v1.41 (Broad Institute, http://broadinstitute.github.io/picard/, (2 November 2024) 2014). Gene-level read counts were generated by featureCounts v1.5.0-p3 [36] and normalized to FPKM. For SNP/Indel calling, sorted bam files were processed following the GATK3 best-practice pipeline [37]. Briefly, SAMtools v0.1.18 [38] was used for index creation, and GATK3 was applied for variant calling with hard filters: clusterWindowSize 10, MQ0 ≥ 4 and (MQ0/DP) > 0.1, QUAL < 10, QUAL < 30.0||QD < 5.0||HRun > 5. Only variants separated by >5 bp were retained. Differential expression analysis between conditions was performed for samples with biological replicates. Differentially expressed genes were identified by initially filtering for *p*-value < 0.05 using a *t*-test, and subsequently applying a |log_2_(FoldChange)| > 1 criterion [39]. KEGG [40] pathway enrichment analysis of differentially expressed genes was conducted using KOBAS 2.0 [41].

### 2.5. Gene Expression Quantification Assay

The fungal colonization levels and the genes of the mTOR signaling pathway in JPS were quantitatively determined by qPCR.

The total genomic DNA of each JPS sample was extracted using a TianNamp Genomic DNA kit (Cat No. DP304, Tiangen, Beijing, China) according to the manufacturer’s protocol and cDNA was synthesized from 1 µg of total RNA (See Section 2.3) using the cDNA Synthesis SuperMix (Cat No. 11141ES60, Yeasen, Shanghai, China) in accordance with the manufacturer’s instructions. Subsequently, quantitative real-time polymerase chain reaction (qRT-PCR) was performed. All the specific primers used in this study are listed in Table S2.

### 2.6. Rapamycin and Torin-1 Treatment

Rapamycin (Cat No. R10006, Psaitong, Beijing, China) was dissolved in DMSO to prepare a stock solution of 1 μg/mL, which was then further diluted with 0.9% NaCl solution to working solutions of 2 ng/mL, 4 ng/mL, and 8 ng/mL. Torin-1 (10 mM in DMSO, Cat No. T10315, Psaitong, Beijing, China) was diluted with 0.9% NaCl solution to working solutions of 100 nM, 200 nM, and 400 nM.

The third-instar larvae of JPS were infected by Bb or Ma as described in Section 2.2 and treated with Rapamycin and Torin-1 treatment at 24 hpi, while the control group was injected with an equal volume of 0.9% NaCl containing the same amount of DMSO as present in the highest-concentration inhibitor working solution. Since the experimental larvae were subjected to both fungal infection and inhibitor injection, in addition to the negative control group for the inhibitor, a blank control group was separately designed for each fungus, receiving only the fungal infection without any injection, to correct for mortality. Corrected mortality (%) = experimental mortality (%) − blank control mortality (%) was used to exclude inter-individual variability in JPS condition. The mortality rate of JPS larvae in each group was calculated at 48 hpi. The larvae were collected and immediately stored at −80 °C for subsequent analysis.

### 2.7. RNA Interference (RNAi) Assay

To obtain the specific dsRNA of JPS mTOR, in vitro transcription was conducted using the T7 RNAi Transcription Kit (Cat No. TR102-02, Vazyme, Nanjing, China) according to the manufacturer’s instructions. The region of JPS mTOR cDNA was selected to synthesize three sets of dsRNA. The dsRNA synthesis kit provided a technical control (dscontrol), whereas ddH_2_O served as a negative control. Templates for in vitro transcription were amplified using gene-specific primers with the T7 polymerase promoter sequence at their 5′ end. Approximately 1 μg of template was used for a 20 μL in vitro transcription reaction. The reaction mix was incubated at 37 °C for 3–4 h, followed by 30 min of DNase I treatment. The primers used in this work are listed in Table S2.

The third-instar larvae of JPS were infected by Bb or Ma as described in Section 2.2 and treated with RNAi treatment at 24 hpi, respectively. Each larva was injected with approximately 30 μg (<5 μL) dsRNA of JPS mTOR into the pronotum by using a 10 μL syringe (Cat No. WLJYQ-10UL/0.26MM, Bolige, Shanghai, China). Thirty JPS individuals were injected per treatment. The mortality rate of JPS larvae in each group was calculated at 48 hpi. The larvae were collected and immediately stored at −80 °C for subsequent analysis.

## 3. Results

### 3.1. Comparative Pathogenicity Dynamics Between Bb and Ma in JPS Larvae

**Figure 1:**
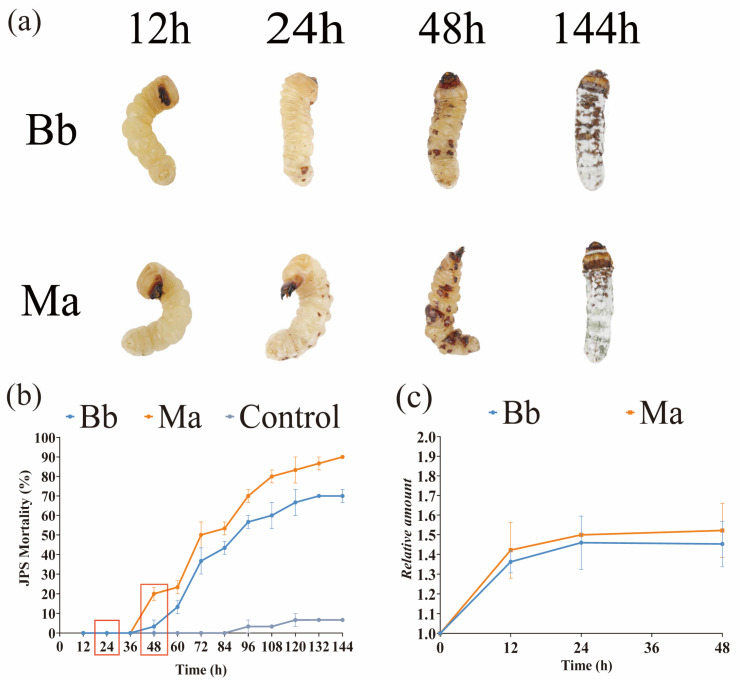
Performance and Mortality Rates of *Monochamus alternatus* (JPS) Larvae Infected with *Beauveria bassiana* (Bb) and *Metarhizium anisopliae* (Ma). (**a**) Phenotypic variations observed in JPS larvae at various time points post-infection with Bb and Ma. (**b**) Mortality trends in JPS larvae following infection with Bb and Ma, red box highlighted the sample time point for further dual RNA-seq. (**c**) Relative fungal biomass within JPS larvae at 12 h, 24 h, and 48 h post-infection with Bb and Ma (Data are presented as mean ± standard error of the mean (SEM) from three independent experiments, each with three replicates).

Our systematic evaluation revealed distinct temporal patterns in the pathogenic progression of *Beauveria bassiana* (Bb) and *Metarhizium anisopliae* (Ma) in JPS larvae (Figure 1). Key pathological manifestations—including cuticular rigidity, body straightening, and fungal sporulation—exhibited strain-specific progression kinetics. Larvae infected with Ma (MaJPS) exhibited accelerated pathogenesis, with visible invasion sites at 24 hpi and pronounced melanization by 48 hpi, whereas those infected with Bb (BbJPS) displayed delayed pathology, with localized invasion becoming evident only at 48 hpi (Figure 1a).

Mortality dynamics revealed a critical inflection point at 48 hpi. Both infected groups maintained 0% mortality during the initial 36 hpi, but significant divergence (*p* < 0.05) emerged thereafter: MaJPS mortality rose to 20% compared to 3.33% in BbJPS at 48 hpi, ultimately reaching 90% and 70% by 144 hpi, respectively (Figure 1b). qPCR-based quantification of fungal biomass indicated that both strains attained comparable relative levels (Bb: 1.45; Ma: 1.52) by 48 hpi (Figure 1c), suggesting that the observed mortality differences reflect strain-specific virulence mechanisms rather than differential proliferation capacity.

### 3.2. Transcriptomic Basis of Differential Host–Pathogen Interactions

**Figure 2:**
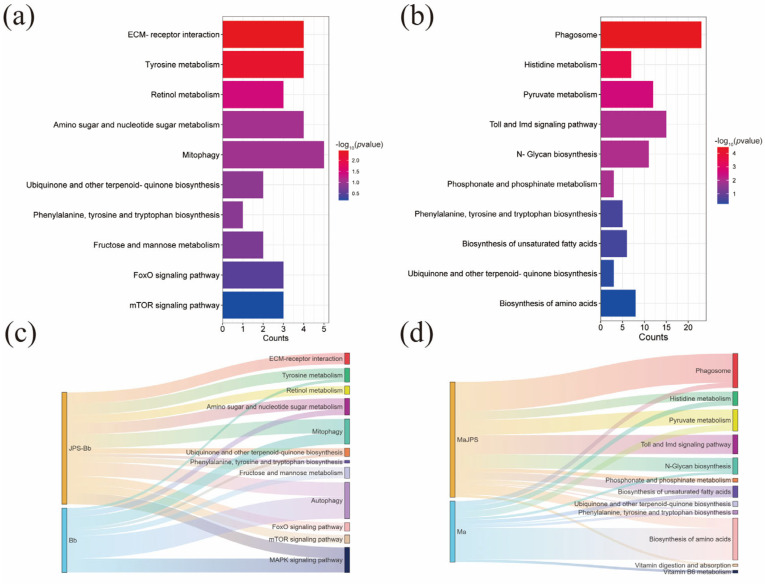
Functional Analysis of Differentially Expressed Genes (DEGs) regard to Host–Pathogen Interactions in JPS Larvae Infected with *Beauveria bassiana* (Bb) and *Metarhizium anisopliae* (Ma). Bar charts illustrating KEGG enrichment analysis of differentially expressed genes in the transcriptome of JPS larvae at 48 h post-infection compared to 24 h for Bb (**a**) and Ma (**b**), displaying enriched metabolic pathways and gene counts; the color gradient corresponds to −log10(*p*-value), with red indicating higher significance (*p* < 0.05, *t*-test). Sankey diagram mapping the pathways implicated in the significantly changed genes of both fungi and larvae at 48 h versus 24 h post-infection with Bb (**c**) and Ma (**d**).

Comparative transcriptomic analysis of JPS larvae infected with Bb or Ma, performed using dual RNA-seq, focused on the critical transition period (48 Hpi Vs. 24 hpi). KEGG enrichment analysis of the dual-species (fungal-insect) transcriptomes revealed pathogen-specific reprogramming of host metabolism and immunity. Specifically, BbJPS showed significant enrichment of pathways related to host tissue degradation and fungal nutrient acquisition, including ECM-receptor interaction, mitophagy, and amino sugar/nucleotide sugar metabolism (Figure 2a). In contrast, MaJPS exhibited marked enrichment of host immune defense pathways, including phagosome formation, Toll/Imd signaling, and N-glycan biosynthesis (Figure 2b). Despite these divergent patterns, shared host stress responses were observed in both infections, with co-enrichment in core metabolic pathways such as phenylalanine, tyrosine, and tryptophan biosynthesis, and ubiquinone and other terpene-quinone biosynthesis (Figure 2a,b, Figures S5 and S6).

**Figure 3:**
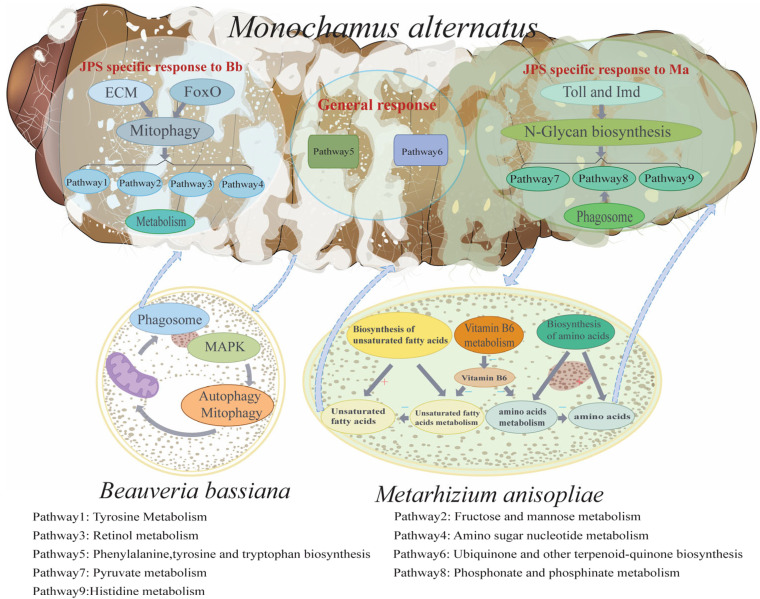
Schematic Diagram Illustrating the Infections of JPS Larvae by Different Fungi. This schematic diagram delineates the comparable and divergent response pathways exhibited by JPS larvae when infected by *Beauveria bassiana* (Bb) and *Metarhizium anisopliae* (Ma). It highlights the shared and unique mechanisms through which the larvae counteract infections from Bb or Ma, as well as the adaptive pathways employed by the fungi in response to the larval host.

Analysis of the integrated fungal transcriptomes within infected hosts highlighted contrasting infection strategies. Bb prioritized autophagy-related pathways and manipulated host sugar metabolism for nutrient acquisition, while simultaneously modulating its pathogenicity via MAPK signaling (Figure 2c and Figure S7). Ma exploited host sugar and amino acid metabolism pathways for biosynthetic processes and adapted through mechanisms involving N-glycan biosynthesis and pyruvate metabolism regulation (Figure 2d and Figure S8). Collectively, these dual-species transcriptomic findings demonstrate the divergent metabolic manipulation and regulatory strategies employed by Bb and Ma during infection. These distinct fungal strategies directly elicited correspondingly distinct host defense responses in JPS larvae (Figure 3). This dynamic interplay underscores the value of interaction transcriptomics in dissecting the molecular mechanisms of fungal invasion and host counter-defense.

### 3.3. Differential mTOR Pathway Regulation Underlies Divergent Mortality Outcomes

**Figure 4:**
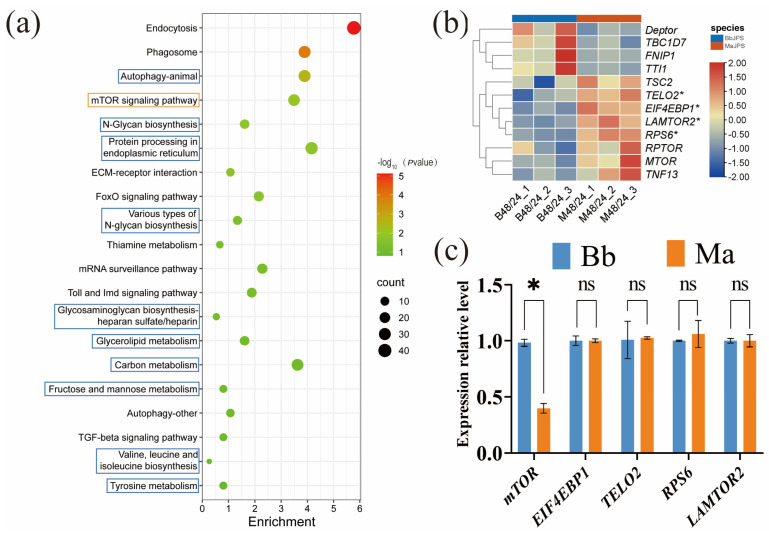
The mTOR Signaling Pathway Exhibits Distinct Responses in JPS Larvae to Infections by *Beauveria bassiana* (Bb) and *Metarhizium anisopliae* (Ma). (**a**) This panel presents the results of a KEGG enrichment analysis on differentially expressed genes in JPS larvae in response to invasions by Bb and Ma (*p* < 0.05, *t*-test). Notably, the mTOR signaling pathway is highlighted in a orange box, while its associated pathways are marked in blue boxes. (**b**) Heatmap visualizes the expression levels of genes involved in the mTOR signaling pathway in JPS larvae following infections by Bb and Ma. The color intensity reflects the degree of gene expression changes. (**c**) The relative quantification results of mTOR-associated genes in JPS larvae at 48 h post-infection with Bb or Ma, as determined by qPCR (Data are presented as mean ± standard error of the mean (SEM) from three independent experiments, each with three replicates). The asterisks (*) indicate significant differences between treatment groups at the *p* < 0.05 level, as determined by one-way ANOVA with multiple comparisons performed using Tukey’s test. The label “ns” denotes non-significant differences (*p* > 0.05).

To elucidate the differential mortality rates of JPS resulting from its distinct responses to Bb and Ma invasion, we first calculated the gene expression ratios (48/24) of Bb-JPS and Ma-JPS at 48 hpi relative to 24 hpi. Subsequently, the ratio of these two ratios (“ratio of ratios”) was computed and used as the background for KEGG enrichment analysis. The mTOR signaling pathway emerged as the most significantly enriched pathway (red highlight, Figure 4a), indicating its pivotal role in host–pathogen interactions. Complementary pathways—including autophagy, N-glycan biosynthesis, and endoplasmic reticulum protein processing (blue highlight)—were co-enriched, suggesting coordinated regulation during infection (Figure 4a).

Transcriptional dynamics of mTOR-associated genes were analyzed via heatmap, comparing JPS infected with Bb or Ma at 48 hpi relative to 24 hpi. Pathogen-specific mTOR modulation was evident from distinct clustering patterns (Figure 4b). Notably, *DEPTOR*, *TBC1D7*, and *FNIP1* were markedly upregulated in MaJPS compared with BbJPS, whereas *TSC2*, *EIF4EBP1*, and *RPS6* exhibited divergent expression patterns. qPCR validation at 48 hpi confirmed these findings: mTOR expression was 2.5-fold higher in BbJPS than in MaJPS (*p* < 0.05; Table S3), consistent with pathway enrichment. Conversely, *EIF4EBP1*, *TELO2*, *RPS6*, and *LAMTOR2* showed no significant differences between the two pathogens (Figure 4c). This differential mTOR activation implies pathogen-specific regulatory mechanisms, potentially linked to Bb’s virulence strategies or immune evasion tactics.

### 3.4. mTOR Signaling Is Critical for Host Defense Against Bb and Exhibits Pathogen-Specific Vulnerability 

**Figure 5:**
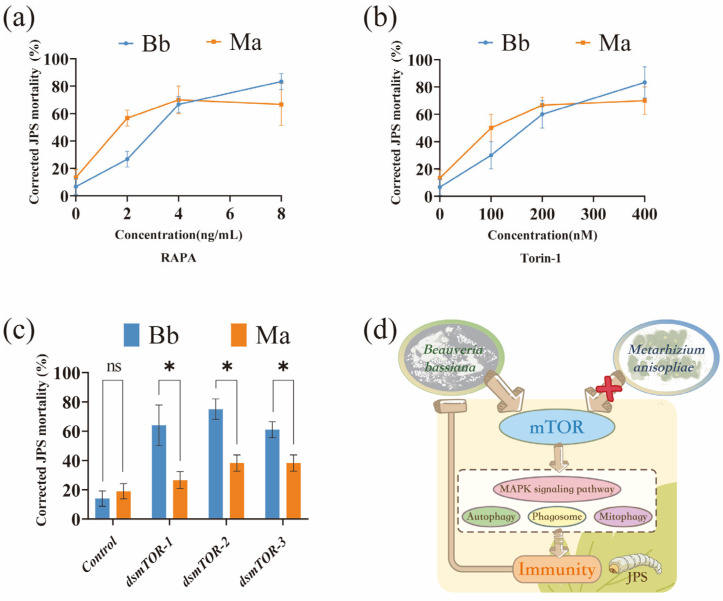
The mTOR signaling pathway plays a crucial role in differential resistance of JPS against *Beauveria bassiana* (Bb) and *Metarhizium anisopliae* (Ma) infection. (**a**–**c**) Corrected mortality of JPS larvae were assessed 24 h post-treatment with: (**a**) rapamycin (RAPA), (**b**) Torin-1, and (**c**) mTOR-targeting RNAi products (*dsmTOR-1*, *dsmTOR-2*, *dsmTOR-3*), following 24 h infections with Bb or Ma. (**d**) Schematic representation of mTOR-mediated immune defense activation upon Bb infection. Data are presented as mean ± SD. Statistical significance (*p* < 0.05, one-way ANOVA with Tukey’s multiple comparisons test) is indicated by “*” (significant) and “ns” (not significant).

To functionally assess the role of mTOR signaling identified in the transcriptomic analysis (Figure 4), we inhibited the pathway pharmacologically (rapamycin, Torin-1) and genetically (RNAi) in JPS larvae infected with Bb or Ma, and evaluated mortality and gene expression (Figure 5 and Figures S9–S11). Pharmacological inhibition of mTOR reversed the baseline mortality pattern (in which MaJPS mortality > BbJPS mortality at 48 hpi). Both rapamycin (RAPA, Figure 5a) and Torin-1 (Figure 5b) induced dose-dependent increases in mortality in infected larvae. At peak concentrations (RAPA: 8 ng/mL; Torin-1: 400 nM), BbJPS mortality (83.3%) significantly exceeded MaJPS mortality (RAPA: 66.7%; Torin-1: 70%; *p* < 0.05), demonstrating that intact mTOR signaling is particularly critical for host survival during Bb infection.

RNAi-mediated silencing of *mTOR* genes (ds*mTOR*-1/2/3) confirmed this pathogen-specific vulnerability (Figure 5c and Figure S11). While control dsRNA groups maintained baseline susceptibility, *mTOR* knockdown consistently caused significantly higher mortality in BbJPS than in MaJPS larvae. Mortality ratios (BbJPS/MaJPS) were 2.4 (ds*mTOR*-1), 1.96 (ds*mTOR*-2), and 1.59 (ds*mTOR*-3) (*p* < 0.05; Table S3), indicating effective suppression and heightened BbJPS dependence on mTOR-mediated defense. This graded effect may reflect silencing efficiency or component-specific roles. Figure 5d integrates these findings, depicting mTOR signaling as a central hub that modulates key antifungal processes, including MAPK signaling, autophagy, phagosome maturation, and mitophagy.

Our results demonstrate that mTOR inhibition disproportionately compromises host defense against Bb compared to Ma. This pathogen-specific vulnerability suggests that Bb’s infection strategy either critically depends on host mTOR activity or exhibits a reduced capacity to evade or compensate for mTOR suppression, potentially reflecting distinct host–pathogen interactions or fungal evasion mechanisms.

## 4. Discussion

EPFs, notably Bb and Ma, are promising biocontrol agents against the Japanese pine sawyer (JPS), the vector of pine wilt disease. However, their variable efficacy and underlying mechanisms are not fully understood, and this knowledge gap limits targeted genetic improvement [42,43]. In this study, we employed dual RNA-seq to dissect the molecular interplay between JPS larvae and these two fungi at a critical infection stage (48 hpi), aiming to identify key determinants of their differential pathogenicity. Our findings revealed the following: (1) Divergent fungal infection strategies. Transcriptomic profiling demonstrated fundamentally distinct infection strategies employed by Bb and Ma. Bb activated autophagy-related processes, the MAPK signaling pathway, and various metabolic pathways, including fructose and mannose metabolism as well as amino sugar and nucleotide sugar metabolism (Figure 2c and Figure 3). In contrast, Ma upregulated amino acid metabolism, fatty acid metabolism, and immune-related pathways, such as biosynthesis of unsaturated fatty acids and the phagosome pathway (Figure 2d and Figure 3). Both fungi co-induced the ubiquinone and other terpenoid quinone biosynthesis pathway. (2) Host mTOR pathway as a response hub. Crucially, our analysis pinpointed the host mTOR signaling pathway as a central hub mediating the differential response of JPS to Bb versus Mainfection (Figure 4a). BbJPS exhibited 2.5-fold higher *mTOR* expression than MaJPS (Figure 4c), and inhibition of mTOR significantly increased BbJPS mortality (Figure 5). Together, these findings comprehensively elucidate the real-time molecular interactions between different EPFs (Bb and Ma) and their host, JPS.

Previous studies of insect–fungus interactions have predominantly focused on single-pathogen systems [11,20,21]. In contrast, our comparative analysis of Bb and Ma infections in JPS larvae revealed distinct pathogen-specific reprogramming of metabolic and signaling pathways (Figure 2). From the perspective of JPS under EPF invasion, BbJPS upregulated tyrosine metabolism and mitophagy (Figure 2a), whereas MaJPS activated pyruvate metabolism and the biosynthesis of unsaturated fatty acids (Figure 2b). These findings diverge from previous insect–fungus interaction studies, which mainly emphasized conserved immune pathways such as Toll/Imd signaling or phenoloxidase activation. Notably, shared pathways such as terpene quinone biosynthesis point to a conserved host detoxification mechanism against fungal secondary metabolites, highlighting the host’s dual strategy of pathogen-specific metabolic reprogramming and broad-spectrum chemical defense [44]. The pathogen-specific metabolic changes observed here suggest that JPS larvae adopt tailored responses according to the pathogenic strategies of the infecting fungi (Figure 2 and Figure 3). From the perspective of EPF pathogenesis, Ma’s rapid induction of pyruvate metabolism may provide energy for accelerated hyphal growth and toxin synthesis [45], such as demethylated toxins, consistent with its early-lethal phenotype (Figure 1a). In contrast, Bb’s activation of tyrosine metabolism—linked to insect melanization and immune signaling—may reflect a stealthier invasion strategy to delay immune detection [46]. These differences may stem from distinct fungal effector molecules (e.g., Bb’s secondary metabolites such as oosporein versus Ma’s proteases) or from evolutionary adaptations in host–pathogen interactions [47].

Our transcriptomic and functional analyses reveal for the first time that JPS larvae combat Bb infection via a unique dual-regulatory strategy. In BbJPS, both the mTOR and FoxO signaling pathways were significantly activated (Figure 2a), and in the analysis of JPS differential responses to Bb versus Ma invasion, these two pathways were also enriched (Figure 4a). This challenges the conventional view of mutual antagonism between mTOR and FoxO in insect immunity. Typically, mTOR inhibits FoxO under nutrient-rich conditions, but in BbJPS their co-activation suggests a pathogen-driven adaptation [48,49]. This adaptation restricts fungal proliferation through mTOR-mediated autophagy while mitigating host-derived oxidative stress via FoxO-regulated antioxidant enzymes [50]. Such dual activation is consistent with Bb’s more subtle infection strategy (Figure 1), which prioritizes immune evasion over rapid tissue destruction. By contrast, Ma infection bypassed mTOR-dependent defenses and instead upregulated histidine metabolism and nitrate assimilation pathways (Figure 3 and Figure 5d), likely to fuel its rapid cuticle degradation and toxin synthesis (e.g., demethylated toxins), further emphasizing the pathogen-specific nature of this regulatory divergence [45]. The absence of FoxO activation in Ma-infected hosts further supports its reliance on alternative pathogenic strategies, such as mechanical penetration and toxin-mediated immune suppression (Figure 2b). Together, these findings redefine the roles of mTOR and FoxO in antifungal immunity: BbJPS simultaneously activates both pathways to balance fungal control with stress alleviation [51].

Despite pathway-level differences, some mTOR-related genes (such as *EIF4EBP1* and *RPS6*) showed no differential expression (Figure 4c). This may reflect post-transcriptional regulation (e.g., phosphorylation) or compensatory feedback loops, as mTOR integrates multiple upstream signals (AMPK, PI3K-Akt) [52]. Potential off-target effects of RNAi could also influence mortality results; however, the graded response across the three dsRNA constructs supports specificity (Figure 5c and Figure S11b,c). To overcome these limitations, proteomic approaches—such as quantitative analysis of phosphorylated mTOR—as well as CRISPR-Cas9–mediated knockout of mTOR in JPS could help clarify the regulatory dynamics [53,54]. In addition, testing alternative inhibitors (e.g., everolimus) and extending the observation period beyond 48 hpi may reveal time-dependent changes in pathway relevance [55]. In this study, Ma displayed faster invasion than Bb, which contrasts with prior research and is likely attributable to the specific Bb and Ma strains used here.

This study addresses a key gap in understanding how coleopteran hosts dynamically rewire their molecular networks to resist phylogenetically distinct fungi. By linking pathogen-specific metabolic changes to mTOR-dependent immunity, our work provides a framework for precision biocontrol [56]. For example, engineering Bb strains to express mTOR inhibitors (such as viral TOR inhibitors) could enhance their pathogenicity, whereas Ma-based biopesticides may benefit from pyruvate-metabolism-targeting adjuvants. In addition, small-molecule mTOR modulators could be developed as JPS-targeted synergists, enhancing fungal lethality without harming non-target species [57]. Combining these strategies with RNAi-based tree protection has the potential to revolutionize PWD management. By leveraging host–pathogen interactions, this approach could reduce reliance on chemical pesticides and mitigate ecological harm [58].

## 5. Conclusions

In this study, we performed dual RNA-seq to systematically compare transcriptional responses between JPS and Bb/Ma during infection, identifying distinct infection strategies. We found that Bb preferentially activated autophagy pathways and modulated host carbohydrate metabolism to facilitate nutrient acquisition, whereas Ma primarily co-opted host amino acid and sugar metabolic pathways for biosynthetic processes. Notably, the mTOR signaling pathway was identified as a key regulator of differential host responses to the two entomopathogenic fungi. Furthermore, functional validation confirmed that mTOR inhibition selectively enhanced Bb-induced mortality of JPS without affecting Ma virulence. These findings reveal the molecular determinants of host–pathogen specificity in PWD biological control and suggest that mTOR regulation could serve as an effective strategy to improve fungal pesticide performance.

## Data Availability

The data that support the findings of this study are available from the corresponding author upon reasonable request. The dataset generated can be found in the Sequence Read Archive (SRA) with the accession number PRJNA1286093. The data that support the findings of this study are available from the corresponding author upon reasonable request prior to public release. The FPKM expression matrix used for all downstream analyses is provided as Supplementary File Tables S4–S6.

## Supplementary Materials

The following supporting information can be downloaded at: https://www.mdpi.com/article/10.3390/insects16101006/s1, Figure S1: Composition of raw sequencing reads in BbJPS after quality control; Figure S2: Composition of raw sequencing reads in MaJPS after quality control; Figure S3: Composition of raw sequencing reads in Bb after quality control; Figure S4: Composition of raw sequencing reads in Ma after quality control; Figure S5: Comparative pathway analysis of JPS responses to fungal infections; Figure S6: Differential pathway enrichment in JPS response to fungal infections; Figure S7: KEGG pathway enrichment analysis of JPS transcriptional responses to Bb infection; Figure S8: KEGG pathway enrichment analysis of JPS transcriptional responses to Ma infection; Figure S9: Dose-responsive modulation of mTOR signaling pathway genes by rapamycin(RAPA) in JPS larvae; Figure S10: Dose-responsive modulation of mTOR signaling pathway genes by Torin-1 in JPS larvae; Figure S11: Functional validation of mTOR-specific RNAi constructs in JPS larvae; Table S1: Sequencing data statistics; Table S2: Primers for qPCR and RNAi experiments; Table S3: Data statistics; Table S4: fpkm_genename-Bb; Table S5: fpkm_genename-Ma; Table S6: fpkm_genename-JPS.
