# Supplementary material for: Comparative Transcriptomics Unveils Pathogen-Specific mTOR Pathway Modulation in Monochamus alternatus Infected with Entomopathogenic Fungi
Source: Insects. 2025 Sep 28;16(10):1006. doi: 10.3390/insects16101006 (PMC12565087; doi:10.3390/insects16101006)
Supplement: Supplementary file 1 [file insects-16-01006-s001.zip › Supplementary Figure.pdf]

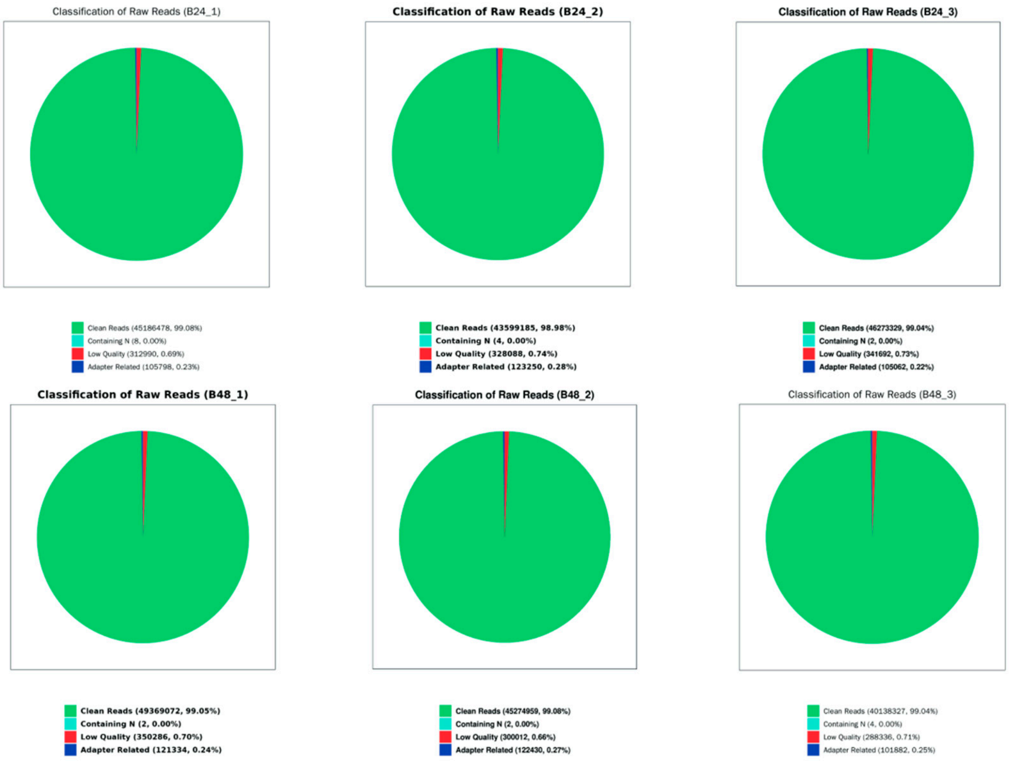

Figure S1. Composition of raw sequencing reads in BbJPS after quality control. *Clean Reads* represent high-quality retained sequences; *Reads with N* were discarded due to ambiguous bases; *Low-Quality Reads* were removed based on Phred score thresholds; *Adapter-Related Reads* were excluded due to adapter contamination.

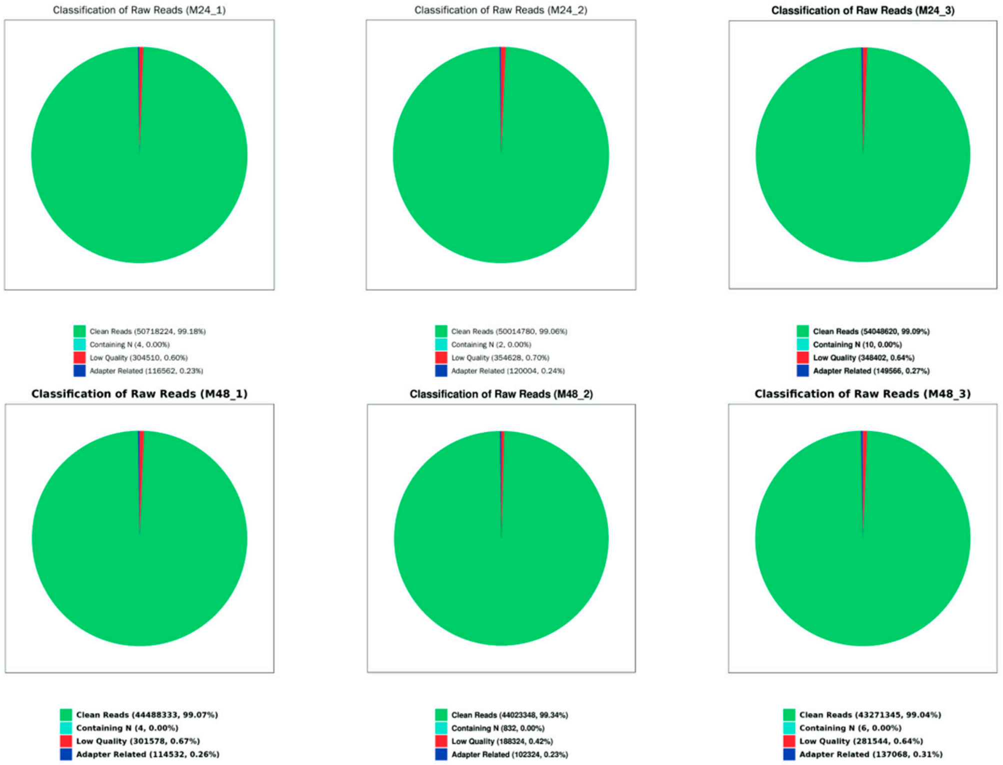

Figure S2. Composition of raw sequencing reads in MaJPS after quality control. *Clean Reads* represent high-quality retained sequences; *Reads with N* were discarded due to ambiguous bases; *Low-Quality Reads* were removed based on Phred score thresholds; *Adapter-Related Reads* were excluded due to adapter contamination.

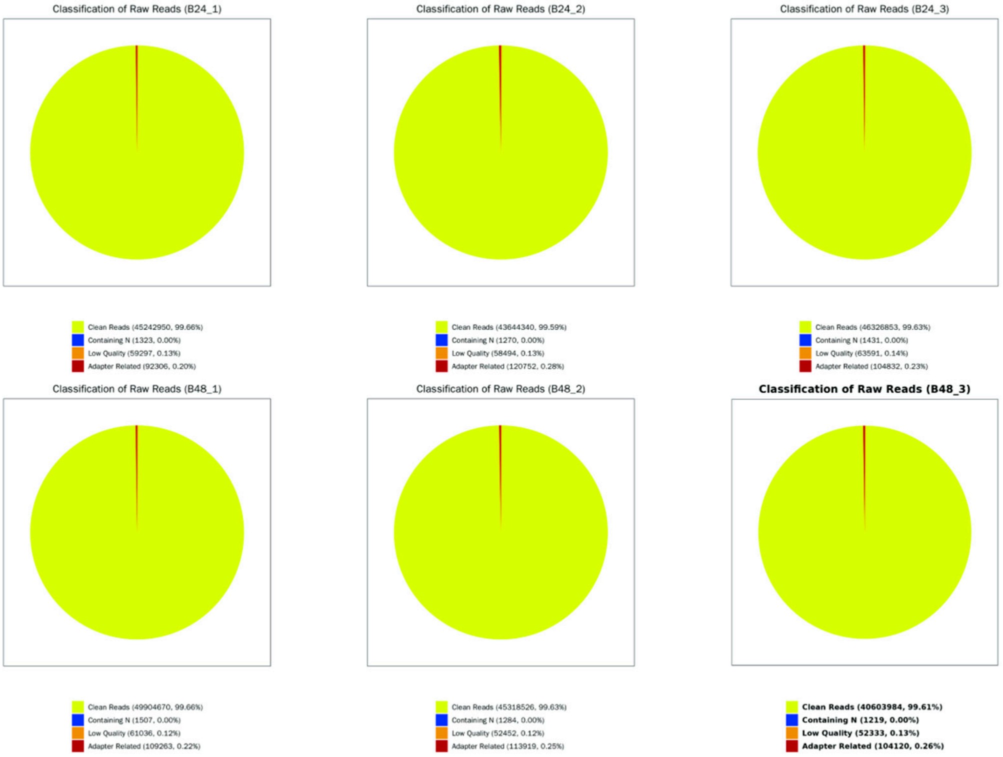

Figure S3. Composition of raw sequencing reads in Bb after quality control. *Clean Reads* represent high-quality retained sequences; *Reads with N* were discarded due to ambiguous bases; *Low-Quality Reads* were removed based on Phred score thresholds; *Adapter-Related Reads* were excluded due to adapter contamination.

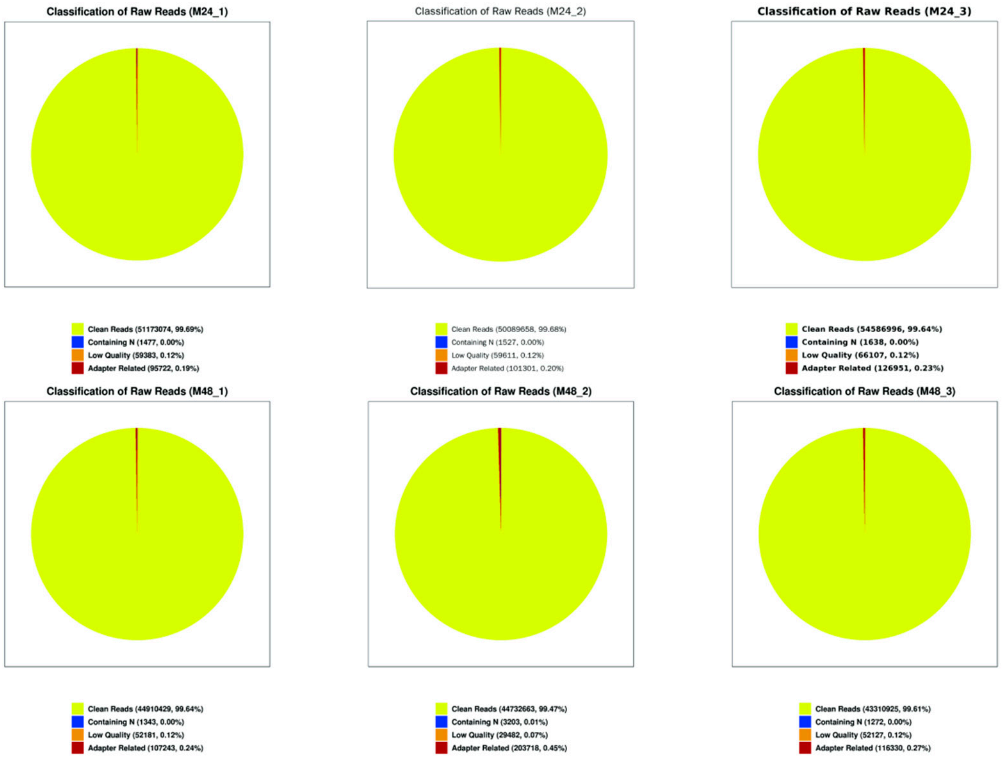

Figure S4. Composition of raw sequencing reads in Ma after quality control. *Clean Reads* represent high-quality retained sequences; *Reads with N* were discarded due to ambiguous bases; *Low-Quality Reads* were removed based on Phred score thresholds; *Adapter-Related Reads* were excluded due to adapter contamination.

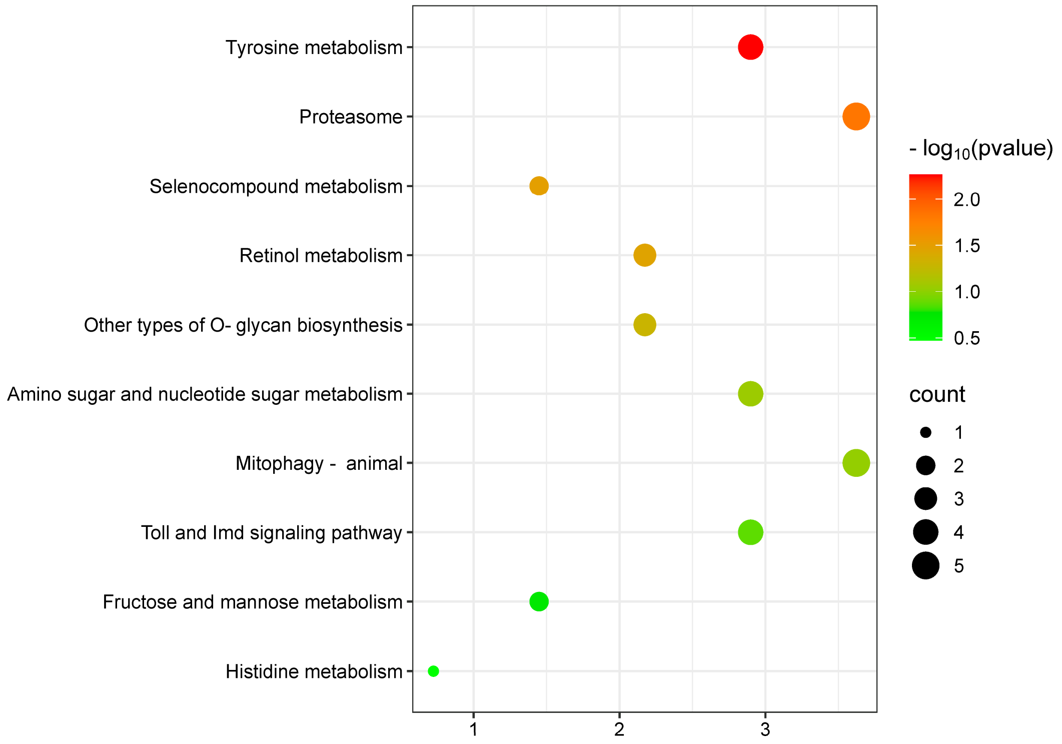

Figure S5. Comparative pathway analysis of JPS responses to fungal infections. Shared metabolic pathways significantly enriched ($p < 0.05$) during both fungal infections include tyrosine metabolism, proteasome activity, selenocompound metabolism, retinol metabolism, and amino sugar/nucleotide sugar metabolism. Bb-specific infection pathways comprise mitophagy, Toll/Imd signaling, fructose/mannose metabolism, and histidine metabolism. Color intensity represents -log10(p-value) (range: 0.5–2.0), with darker shades indicating greater statistical significance.

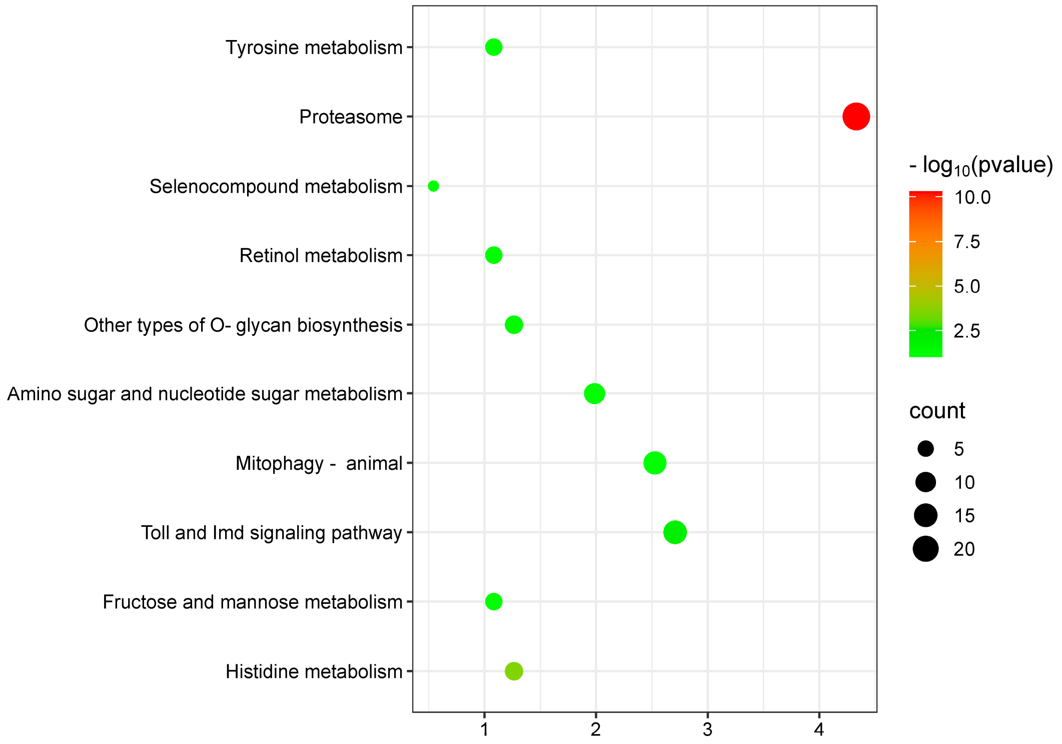

Figure S6. Differential pathway enrichment in JPS response to fungal infections. Shared response pathways ($p < 0.05$) include tyrosine metabolism, proteasome activity, selenocompound metabolism, and amino sugar/nucleotide sugar metabolism. Ma-specific response pathways comprise fructose/mannose metabolism, histidine metabolism, and Toll/Imd signaling. The color gradient represents −log10(p-value) (range: 2.5–10.0), with increasing intensity corresponding to higher statistical significance.

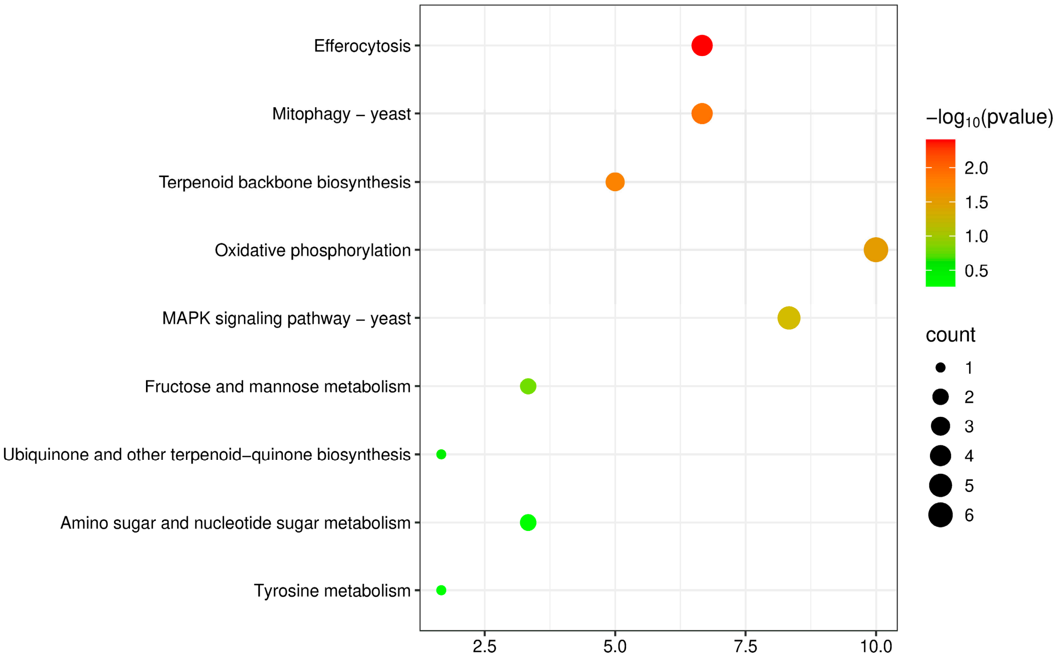

Figure S7. KEGG pathway enrichment analysis of JPS transcriptional responses to Bb infection. Pathway analysis revealed Bb's preferential activation of autophagy-related pathways and host sugar metabolism remodeling for nutrient acquisition, coupled with virulence regulation through MAPK signaling. Color gradient represents -log10(p-value) (range: 0.5–2.0), with increasing intensity indicating greater statistical significance.

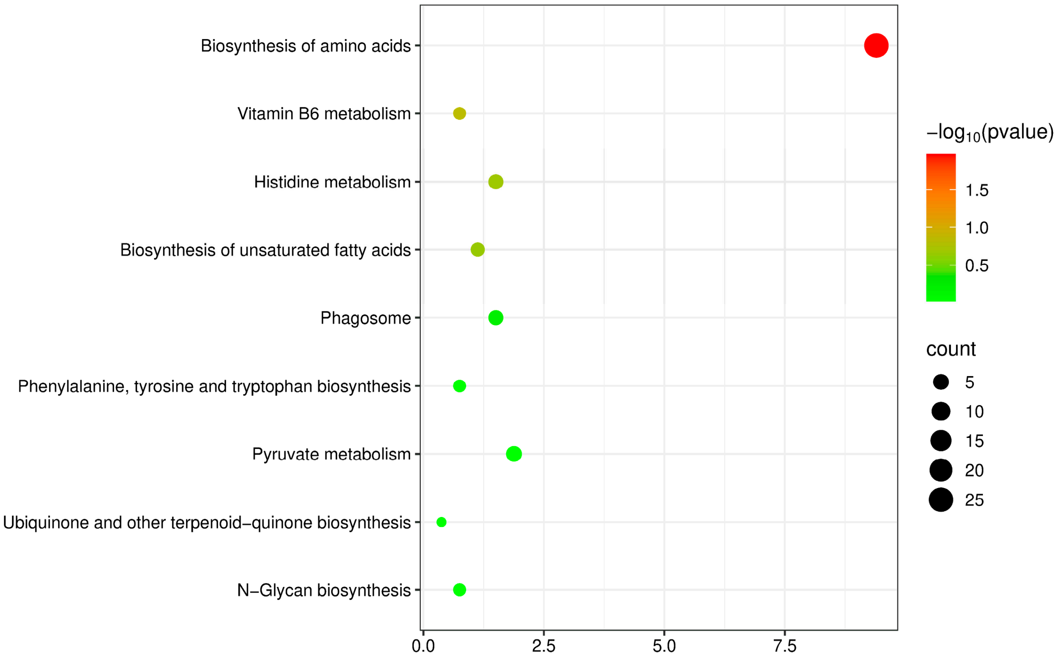

Figure S8. KEGG pathway enrichment analysis of JPS transcriptional responses to Ma infection. The analysis revealed Ma's preferential utilization of host sugar and amino acid metabolism pathways to support biosynthetic demands, while employing adaptive strategies through N-glycan biosynthesis and pyruvate metabolism regulation.Color gradient represents -log10(p-value) (range: 0.5–1.5), with increasing intensity indicating greater statistical significance.

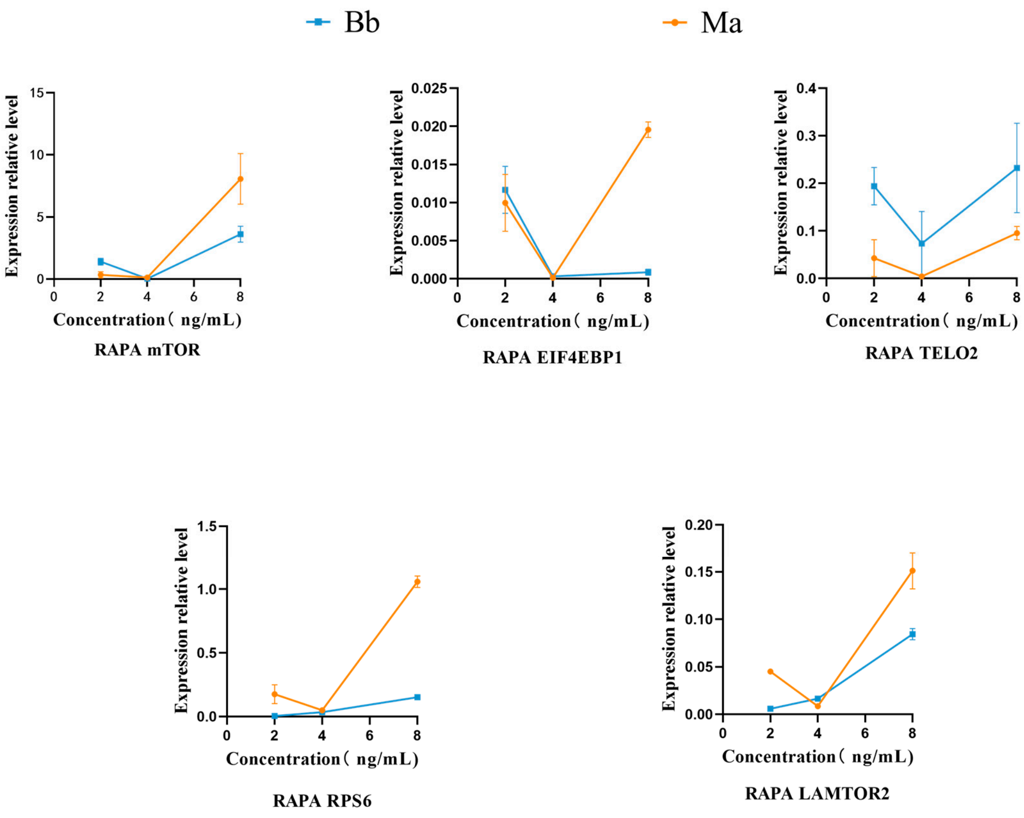

Figure S9. Dose-responsive modulation of mTOR signaling pathway genes by rapamycin(RAPA) in JPS larvae. qPCR analysis of mTOR pathway components (mTOR, EIF4EBP1, and LAMTOR2) following treatment with increasing rapamycin concentrations (0-8 ng/mL). Gene expression is normalized to untreated controls. Dose-response profiles revealed progressive suppression of mTOR and LAMTOR2 transcription at higher concentrations, contrasting with the biphasic regulation of EIF4EBP1. These findings demonstrate concentration-dependent pharmacological inhibition of mTOR signaling in JPS larvae, with component-specific regulatory patterns. Data are presented as mean ± standard error of the mean (SEM) from three independent experiments, each with three replicates.

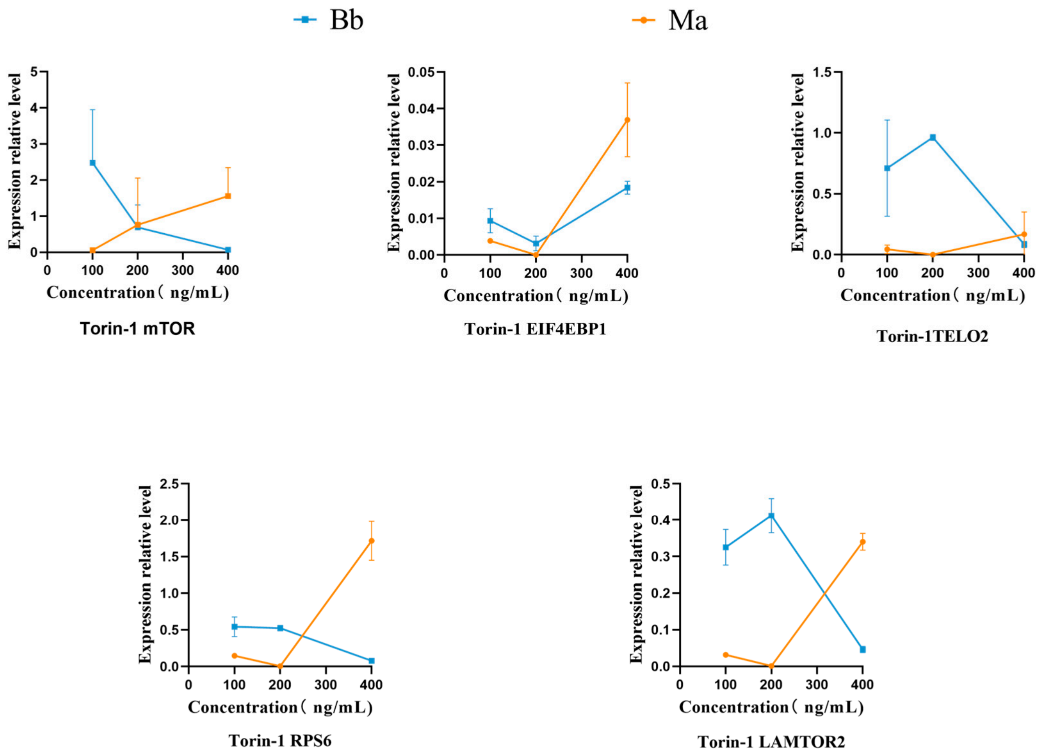

Figure S10. Dose-responsive modulation of mTOR signaling pathway genes by Torin-1 in JPS larvae. qPCR analysis of mTOR pathway components (mTOR, EIF4EBP1, and LAMTOR2) following treatment with increasing Torin-1 concentrations (0–400 nM). Gene expression was normalized to untreated controls. Dose-response profiles revealed progressive transcriptional suppression of mTOR and LAMTOR2, while EIF4EBP1 exhibited a biphasic response pattern, indicating component-specific pharmacological sensitivity to mTOR kinase inhibition. Data are presented as mean ± standard error of the mean (SEM) from three independent experiments, each with three replicates.

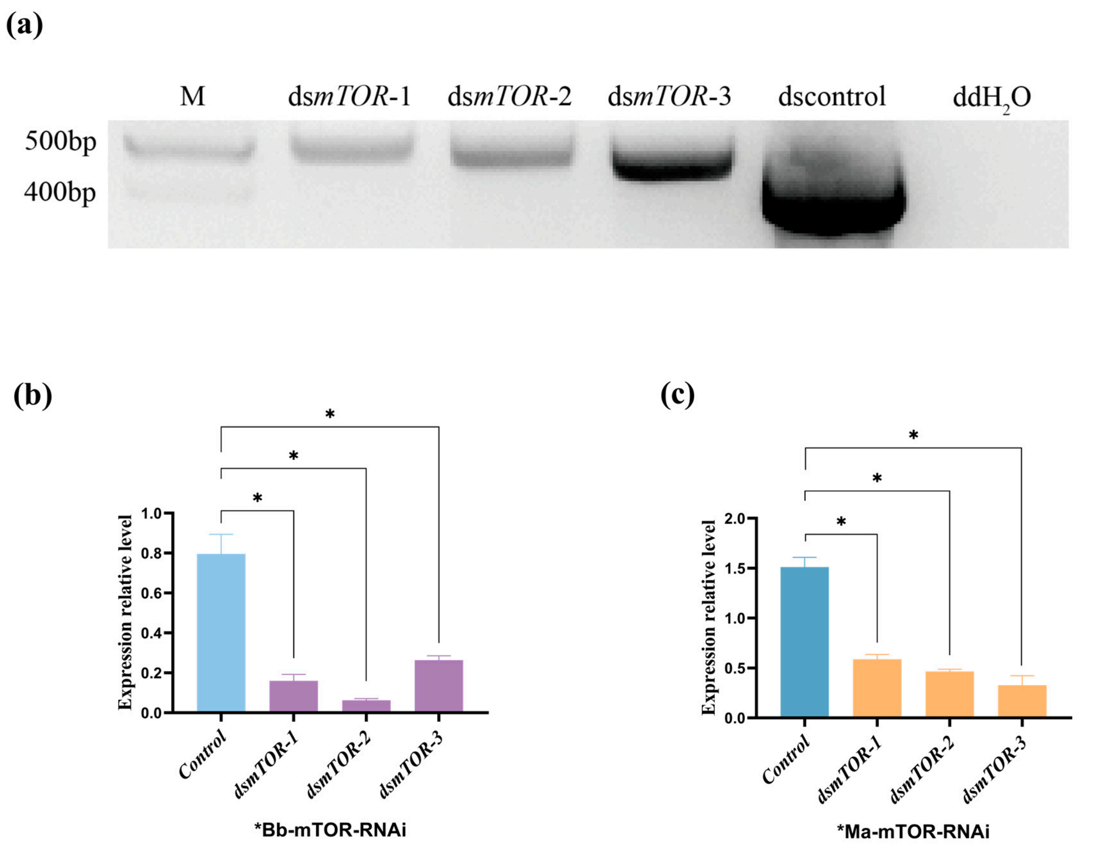

Figure S11. Functional validation of mTOR-specific RNAi constructs in JPS larvae. (**a**) Agarose gel electrophoresis analysis of *mTOR*-specific dsRNA constructs (ds*mTOR*-1: ~500 bp; ds*mTOR*-2/3: ~400 bp). Lanes: M (DNA ladder), ds*mTOR*-1, ds*mTOR*-2, ds*mTOR*-3 (targeting distinct *mTOR* regions), control (non-targeting dsRNA), and ddH_2O (negative control). qPCR analysis of *mTOR* transcript levels in (**b**) BbJPS and (**c**) MaJPS larvae following RNAi treatment. All three dsRNAs significantly suppressed *mTOR* expression ($p < 0.05$, t-test) compared to both negative controls, with dsmTOR-1 demonstrating maximal silencing efficiency (~X-fold reduction). Data are presented as mean ± standard error of the mean (SEM) from three independent experiments, each with three replicates.
